# Accuracy of intraoral scans in the mixed dentition: a prospective non-randomized comparative clinical trial

**DOI:** 10.1186/s13005-020-00222-6

**Published:** 2020-05-19

**Authors:** Konrad Liczmanski, Thomas Stamm, Cristina Sauerland, Moritz Blanck-Lubarsch

**Affiliations:** 1 Private practice, Noldestraße 5, 42551 Velbert, Germany; 2 Department of Orthodontics, University of Muenster, Albert-Schweitzer-Campus 1, 48149 Muenster, Germany; 3 Institute of Biostatistics and Clinical Research, University of Muenster, Schmeddingstraße 56, 48149 Muenster, Germany

**Keywords:** Intraoral scan, Accuracy, Digital, Orthodontics

## Abstract

**Objective:**

In-vivo accuracy of intraoral scans of complete mixed dentitions of patients in active treatment have not yet been investigated. The aim was to test the hypothesis that dimensional differences between intraoral scans and conventional alginate impressions in the mixed dentition are clinically irrelevant.

**Methods:**

Trial design: Prospective non-randomized comparative clinical trial. Based on sample size calculation 44 evaluable mixed dentition jaws of patients in active orthodontic treatment were included. Each patient received an alginate impression following an intraoral scan (TRIOS® Ortho). Plaster cast was fabricated and scanned with an external scanner (ATOS-SO®). Both STL datasets were analyzed with the 3D inspection and mesh processing software GOM Inspect®. Statistical analysis comprised sample size calculation, t-test as well as nonparametric tests.

**Results:**

The absolute mean difference between digital plaster casts and intraoral scans is 0.022 mm ± 0.027 mm (median 0.015 mm). The obtained measurements are in the range of comparable studies on full arch permanent dentitions. Gender, the size of the jaw represented by the dentition stage and upper respectively lower jaw, as well the malocclusion have no effect on the total deviations between digital plaster casts and intraoral scans. Detectable impression errors were bubbles in fissures and marginal ridges as well as incomplete alginate flow and detachment from the tray. Detectable scanning errors were incomplete distal surface of the most distal molar.

**Conclusion:**

Dimensional differences between intraoral scans and conventional alginate impressions in the mixed dentition are clinically irrelevant for orthodontic purposes. In all clinical situations of active treatment in the mixed dentition, the intraoral scans are more detailed and less error-prone.

## 1.Introduction

Alginate is one of the most frequently used dental materials [1]. The low cost, the good tolerability by younger patients, the ease of handling, the short setting time, the simple technique and the sufficient precision [2] makes alginate the gold standard for orthodontic diagnostics and manufacturing removable appliances.

**Fig. 1:**
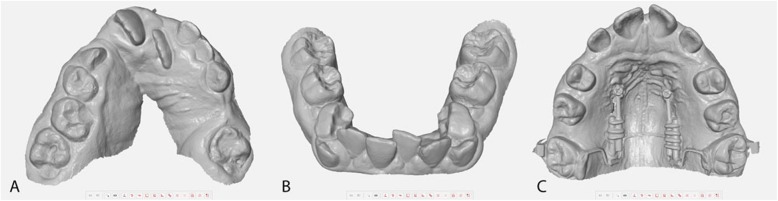
Examples of challenging situations for alginate impressions in the mixed dentition. **a**) Palatal cleft **b**) Crowding with several eruption problems **c**) Bands, wires, micro screws with distalisation device

With the ongoing development of digital procedures, intraoral scanning devices and associated workflows are conquering the dental practice. The intraoral scan and its digital models are being considered as a replacement for conventional impressions in orthodontics because of several potential advantages in hygienic handling, comfort of treatment, transferring of data, analysing and storaging diagnostic models and manufacturing orthodontic appliances. However, both techniques, the conventional alginate impression and the intraoral scan, have different advantages and disadvantages, so that currently no technique can be preferred for routine orthodontic purposes. The currently high acquisition costs and/or the dependency on closed systems with complex license agreements are hindering many clinicians from purchasing intraoral scanner. In addition to cost-effectiveness, the added value for the patient in terms of accuracy and improvement of clinical outcome also counts. With regard to the manufacture of removeable appliances, the scanning process must be robust against several intraoral conditions (Fig. 1), e.g. saliva-covered surfaces (reflectivity), moving soft tissues, varying object to sensor distances in regions with clefts, missing teeth or eruption problems, different dental materials with different reflectivity (fillings, temporary crowns, brackets) and dimensions (wires, temporary anchorage devices).

The literature provides several comparisons of accuracy which are not reflecting natural tissues and scan sizes which have an effect on accuracy [3]. However, current in-vivo studies are split in their conclusion whether full arch scans are clinically superior [4], equivalent [5] or inferior [6] compared to impressions. To the best of our knowledge, in-vivo accuracy of intraoral scans of complete mixed dentition in active treatment have not yet been investigated. The aim of the study was therefore to compare intraoral scans with their respective digital plaster casts in children with mixed dentition. The null hypothesis was that dimensional differences between intraoral scans and conventional alginate impressions in the mixed dentition are clinically irrelevant.

## 2.Material and methods

### 2.1.Trial design

The present study was a prospective non-randomized comparative clinical trial conducted at the University Clinic of Muenster, Germany. Differences between digital models from alginate impressions and intraoral scans of orthodontic patients were investigated. Approval for conducting the study was received from the Ethics Committee of the Medical Faculty of the University of Muenster (2013–603-f-S).

### 2.2.Participants

Eligibility criteria were as follows: (i) orthodontic patients in active treatment (ii) patients in the early or late mixed dentition phase and (iii) patients who will receive an alginate impression for the construction of a removable orthodontic appliance. Exclusion criteria were as follows: (i) handicapped patients with oral sensomotory discomfort and (ii) patients with restricted mouth opening capabilities. Patients were recruited sequentially for impression-taking appointments, regardless of the type of device planned. Eligibility determination as well as the intervention took place at the Department of Orthodontics, the University Clinic of Muenster, Germany.

### 2.3.Interventions

A written informed consent was obtained from each participating child and its legal guardian prior to the start of the study. At the following appointment an intraoral scan was performed with the TRIOS® Ortho (3Shape, Copenhagen, Denmark) prior to the alginate impression. All scans were made by investigator KL. Calibration and scanning process were performed following the instructions of the manufacturer. Alginate impression and plaster (Tetrachrom®, Kaniestone®, Kaniedenta, Herford, Germany) model fabrication were routinely done by the staff of the orthodontic department and dental laboratory. It was ensured that the impressions were stored in humid conditions during transport and that they were poured after 40–60 min. Prior to the construction of the removable appliance the plaster models were scanned with an ATOS-SO® system (GOM GmbH, Braunschweig, Germany), a unit for measuring volumes up to 45 mm × 36 mm × 20 mm with point spacings of 0.03–0.15 mm.

### 2.4.Data handling and processing

The digital models of both systems were exported in standard tessellation language (STL) format for further analysis. To assess differences between the intraoral scan and the scanned plaster cast both STL datasets were imported in GOM Inspect® (GOM GmbH, Braunschweig, Germany), a three-dimensional inspection and mesh processing software.

With GOM Inspect® the scan of the plaster cast was imported as a nominal CAD element and served as the reference model. The corresponding mesh data from the intraoral scan was then imported as an actual data element and was set as the test model. During alignment the nominal data was locked in its position where the actual data were aligned to it. Bringing the two datasets together an initial alignment were performed prior to inspection. This pre-alignment which included best fit alignment over all data aligns the actual data independently by its start position.

The pre-alignment includes all data and this concerns also the different boundaries of the scanned plaster model and the intraoral scan which may result in positioning errors. Therefore the models were trimmed at the margins and the maximum deviation was set to 3 mm, because no larger deviations in the field of teeth and attached gingiva were measured. A local best fit was performed by selecting a sufficiently large surface area of the actual data and creating a new alignment.

This first inspection was performed with surface comparison on CAD where the software calculates the perpendicular distance of each polygon point on the CAD data to the actual data. The software displays the deviation as a color plot on a copy of the CAD data. The colors represent measuring data above and below the CAD surface.

The color mesh of deviations is an overview of the entire alignment and the GOM Inspect® software can flag particular colored areas which could be subjectively selected. To avoid operator selected deviations the entire dataset was analysed. For this reason the geometry of the surface comparisons was exported as an ASCII file with the following parameters: default unit (mm); deviation x/y/z; total deviation.

### 2.5.Statistics

Statistical analyses were performed using IBM SPSS Statistics version 25 (IBM Corp., New York, United States). Based on a pilot study sample size calculation was performed under the assumption of a mean difference of 0.067 mm and a standard deviation of 0.0868 mm between both digital models. The interval for the equivalence bounds for the mean was [0.1 mm +/− 0.1 mm]. It was assumed that the difference is normally distributed, therefore an equivalence test for paired mean difference with a desired significance level of 0.05 was used. Based on this information and a correlation of r = 0.6 between both methods, the necessary sample size comprised 31 evaluable digital models to detect relevant differences with 80% statistical power.

The data were described by frequencies, mean, standard deviation, median, minimum and maximum. To compare the total deviation between lower and upper jaw a t-test for paired means was used, scan time and number of polygon point were analyzed using the Wilcoxon test. Total deviations in gender, dentition phase and angle class were analysed using unpaired t-tests or analysis of variance. Statistical comparisons for scan time and number of polygon points were performed using Mann-Whitney U test or the Kruskal-Wallis test. All reported *P* values were two-sided, *P* values less than 0.05 were considered significant.

## 3.Results

Twenty-six patients (16 female, 10 male) were recruited for the study. All patients were in active treatment and a removable appliance was planned according to their treatment plan. Due to lack of time one patient could not go through the intraoral scan, two patients did not receive an alginate impression and in two cases only one jaw was impressed, so that in total 44 jaws were analysed (Table 1). Mixed dentition phase and the corresponding Angle class are shown in Table 2. 
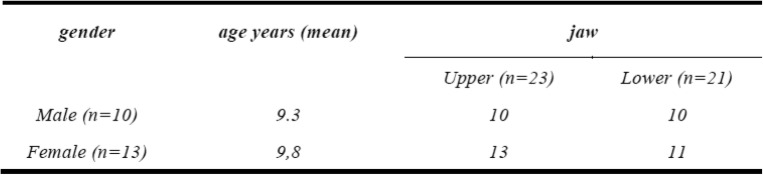

**Table 1:** Age and gender distribution of included patients

Gender	Age years (mean)	Jaw	
		Upper (*n* = 23)	Lower (*n* = 21)
Male (*n* = 10)	9.3	10	10
Female (*n* = 13)	9.8	13	11


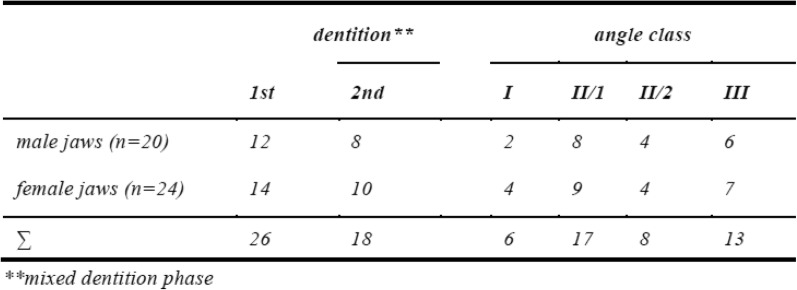

**Table 2:** Obtained variables of the included jaws

	Dentition^a^		Angle class			
	1st	2nd	I	II/1	II/2	III
Male jaws (*n* = 20)	12	8	2	8	4	6
Female jaws (*n* = 24)	14	10	4	9	4	7
∑	26	18	6	17	8	13

^a^mixed dentition phase

There is no difference between males and females concerning the jaws analysed (Table 1), therefore no further subgroup analyses were made. The majority of patients had an Angle class II division 1 malocclusion followed by a class III, class II division 2, and class I. The class I comprises open bites or arches with crowding and transverse or mesio-distal discrepancies (Table 2).

The scan time shows no differences between gender, dentition phase, jaw or Angle class (Table 3). Only the amount of data measured in number of polygon points between upper and lower jaw are different (*p* < 0.001). 
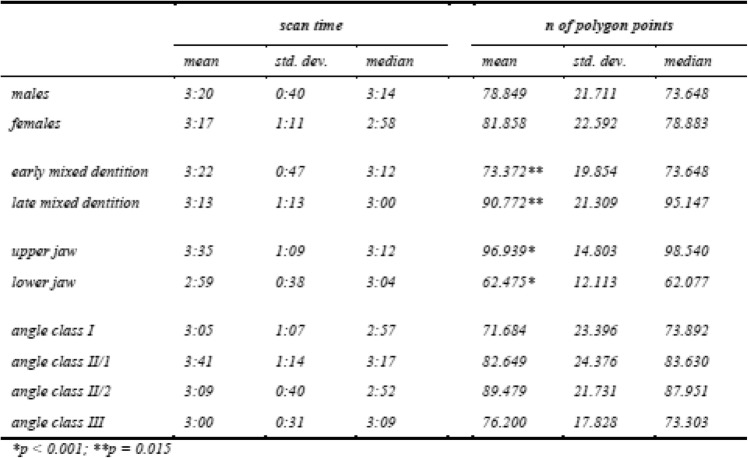

**Table 3:** Scan time (minutes:seconds) and amount of data (number of polygon points) concerning different patient variables. (detailed *p* values are available in supplementary table)

	Scan time			n of polygon points		
	mean	std. dev.	median	mean	std. dev.	median
Males	3:20	0:40	3:14	78.849	21.711	73.648
Females	3:17	1:11	2:58	81.858	22.592	78.883
Early mixed dentition	3:22	0:47	3:12	73.372**	19.854	73.648
Late mixed dentition	3:13	1:13	3:00	90.772**	21.309	95.147
Upper jaw	3:35	1:09	3:12	96.939*	14.803	98.540
Lower jaw	2:59	0:38	3:04	62.475*	12.113	62.077
Angle class I	3:05	1:07	2:57	71.684	23.396	73.892
Angle class II/1	3:41	1:14	3:17	82.649	24.376	83.630
Angle class II/2	3:09	0:40	2:52	89.479	21.731	87.951
Angle class III	3:00	0:31	3:09	76.200	17.828	73.303

*p < 0.001; **p = 0.015

The exported geometries from GOM Inspect® represent the deviation on x/y/z axis and the total deviation between scanned plaster cast and intraoral scan. These distances could be positive or negative depending on whether the mesh point lies above or below the CAD body point. The absolute mean difference between digital plaster casts and intraoral scans is 0.022 mm ± 0.027 mm (median 0.015 mm). Gender, the size of the jaw represented by the dentition stage and upper respectively lower jaw, as well the malocclusion have no effect on the total deviations between digital plaster casts and intraoral scans (Table 4). Seven out of 44 comparisons show extreme values (outliers with mean values greater than 0.04 mm or less than − 0.04 mm) concerning the different patient variables. Those models were manually inspected and the source of error was assessed. 
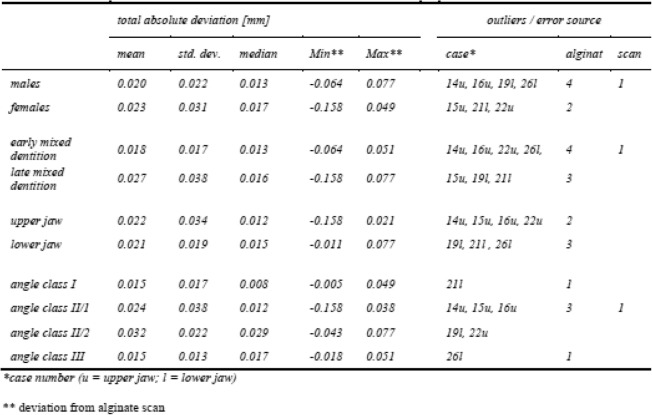

**Table 4:** Descriptive statistics calculated from exported geometries of the surface comparisons. There are no differences in the total absolute deviations (mm) between digital plaster casts and intraoral scans concerning the different patient variables. Cases with extreme values (outliers with mean values greater than 0.04 mm or less than −0.04 mm) were manually inspected and the source of error was assed

	Total absolute deviation [mm]					Outliers / error source		
	mean	std. dev.	median	Min^b^	Max^b^	case^a^	alginate	scan
Males	0.020	0.022	0.013	−0.064	0.077	14u, 16u, 19l, 26l	4	1
Females	0.023	0.031	0.017	−0.158	0.049	15u, 21l, 22u	2	
Early mixed dentition	0.018	0.017	0.013	−0.064	0.051	14u, 16u, 22u, 26l,	4	1
Late mixed dentition	0.027	0.038	0.016	−0.158	0.077	15u, 19l, 21l	3	
Upper jaw	0.022	0.034	0.012	−0.158	0.021	14u, 15u, 16u, 22u	2	
Lower jaw	0.021	0.019	0.015	−0.011	0.077	19l, 21l , 26l	3	
Angle class I	0.015	0.017	0.008	−0.005	0.049	21l	1	
Angle class II/1	0.024	0.038	0.012	−0.158	0.038	14u, 15u, 16u	3	1
Angle class II/2	0.032	0.022	0.029	−0.043	0.077	19l, 22u		
Angle class III	0.015	0.013	0.017	−0.018	0.051	26l	1	

^a^case number (*u* upper jaw, *l* lower jaw)

^b^deviation from alginate scan

**Fig. 2:**
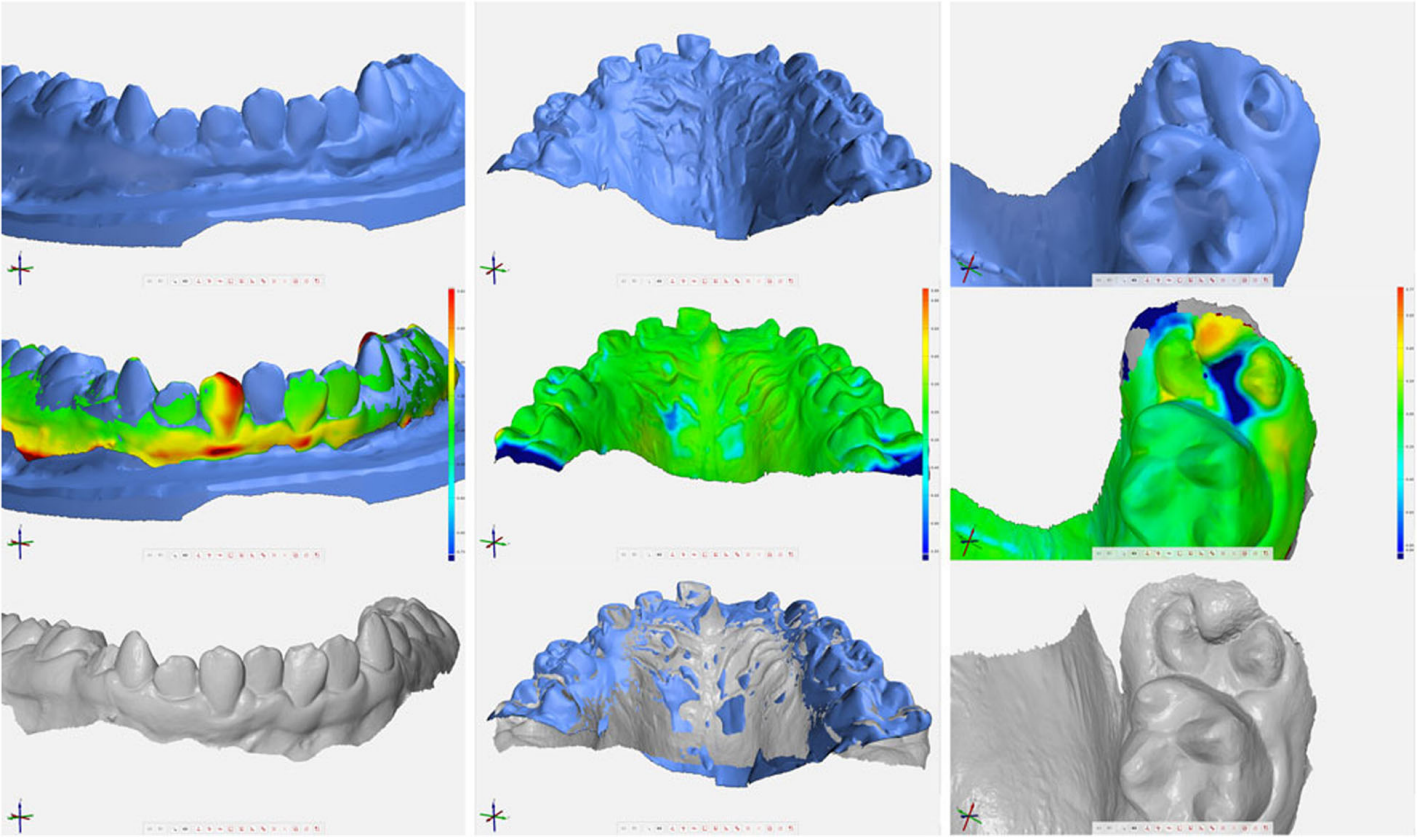
Three examples of errors between plaster cast and intraoral scan. Left column (case 26l): Artifacts of tooth shape which may arise from chemical bonds between hydroxylapatite and alginate. Middle column (case 16u): Relief of the tray during incomplete setting of the alginate could result in distortions of teeth. Right column (case 19u): Incomplete flow of alginate pretends gingival coverings which does not exist. [scanned plaster cast in blue; colored deviation mesh; intraoral scan in grey]

Of the manually inspected models, all errors refer to the alginate impression, with the exception of case 14u, an intraoral scan of an upper jaw with incomplete palatal surface of the upper right dentition (Table 4). Major errors are bubbles in fissures and marginal ridges as well as incomplete alginate flow and detachment from the tray (Fig. 2: 26l, 16u, 19u). Alginate radicals form chemical bonds with enamel hydroxyapatite crystals which could lead to defects in the impression [1]. Shape artifacts may arise from these kind of errors (Fig. 2).

One possible limitation for intraoral scans in the mixed dentition may be the size of the scanner head. Due to the conic shape of the head it could be difficult to access posterior molar areas in adolescents’ with a small oral cavity. Six (13.6%) out of 44 scanned jaws has either on the left or on the right side one incomplete distal surface of the most distal molar. Four (9.1%) had one incomplete surface on both sides.

## 4.Discussion

The clinical accuracy of intraoral scans in the mixed dentition has not yet been investigated. The present study aims to close the gap in research on the routine use of intraoral scans in orthodontic practice. The null hypothesis that dimensional differences between intraoral scans and conventional alginate impressions in the mixed dentition are clinically tolerable could be confirmed. The measured differences of the present study are in the range of measurements published on full arch permanent dentitions. Although several studies reported statistical differences in trueness and precision of intraoral scans, the authors rated the clinical accuracy as equal or higher compared to alginate, which is in accordance with our own results. It is of particular emphasis that this outcome is only valid for the TRIOS® Ortho intraoral scanner.

Unlike to prosthetic dental impressions where silicone or modern polyethers are being used we focused on orthodontic procedures. Especially for children the use of alginate is practicable because of the easy handling and the short processing time. In our study we used alginate which adher to DIN EN ISO 21563 and a class III gypsum. This manufacturing process of plaster casts is general tolerable practice in daily routine for orthodontic purposes but leads to deviations due to application errors which can lead to shrinking/ expansion processes of the plaster casts. In this study those plaster casts were scanned using the ATOS SO scanner which can lead to a maximum deviation of 6.5 μm according to information by the scanner manufacturer. Using a dental impression scanner and comparing its outcome to the IOS models or printing models based on the IOS and compare them with plaster casts are other ways to examine the accuracy of an IOS. We decided against these methods because we wanted to match the current gold standard using alginate and gypsum for orthondontic purposes with the IOS and its digital models excluding inaccuracies produced by the dental impression scanner or by the dental 3D printer.

The question of whether digital models influence the diagnostic decision of orthodontists arises at the beginning of dental plaster cast scanning. Zilberman et al. [7] found that digital models have a lower accuracy than plaster casts when measured tooth size and arch dimensions in various types of malocclusion. Stevens et al. [8] evaluated Bolton- and Peer Assessment Rating measurements in different categories of malocclusions. The authors found no differences in digital versus conventional plaster casts. However, both studies were only able to assess the effect of the digital workflow and handling, since no intraoral scan was used and only duplicated plaster models were scanned.

With the general availability of intraoral scanning devices the full arch permanent dentition scanned under clinical condition became a focus in orthodontic research. The devices were seen as a clinically acceptable alternative to plaster casts and calipers [9] and valid to obtain measurements for diagnostic purposes [10, 11].

Before the intraoral scan becomes the new gold standard for orthodontic purposes, it must deliver high accuracy in all clinical situations. Brackets, bands and wires can result in lower accuracy compared to a natural full permanent dentition. An in-vitro study showed that not only the scanning procedure but also the placement of brackets (lingual or buccal) has a significant effect on arch dimensions [12]. The error exceeds up to 2 mm which is clinically meaningful. It must be considered that the error increases in clinical situation with limitation of scanning directions due to restricting oral structures.

In-vivo studies including full arch natural teeth found clinically acceptable differences between alginate impressions and intraoral scans. Compared with polyether and vinylsiloxanether impression materials intraoral scans are equal or less precise but achieve higher precision compared to alginate [13].

Zhang et al. [5] found an average surface difference of 0.1 mm ± 0.03 mm for both jaws which is higher than our values (0.02 mm ± 0.03). It could be speculated that the technical advancement of the TRIOS® Ortho IOS contributes to the better results in the present study.

Concerning the in-vivo reproducibility Zimmermann et al. [14] found errors of 0.16 ± 0.07 mm for alginate and 0.07 ± 0.04 mm respectively 0.09 ± 0.05 mm for two different intraoral scanner. Although the methodology used differs from the present study, the clinical accuracy corresponds to our own observation.

Digital models could also be extracted from cone beam computed tomography (cbct). In patients who undergo orthognathic surgery the segmented dentition would replace intraoral scans or conventional impressions. Jose et al. [15] has shown that dental measurements on segmented models obtained from cbct are less precise than measurements from intraoral laser scanned models. However, with an interobserver error of less than 0.97 mm on cbct models the authors rated their measurements as clinical acceptable.

Despite clinical good accuracy of intraoral scans, knowledge about the source of possible errors is of crucial importance. Although no specific pattern of errors could be found in the literature, more horizontal deviations in the posterior region were reported. Lee et al. [16] compared two scanning devices in-vivo and found highest values of 0.15 ± 0.17 mm and 0.15 ± 0.07 mm respectively in the region of lower first molars. This corresponds with the findings of [4, 5, 17, 18] who reported a horizontal expansion in the posterior regions of the jaws. Different explanations are available. Scanning distortion and centrifugal expansion is explained by the limitation to reach posterior regions with the scanner head [16]. Also the used scanning strategy [19] and incorrect software processing with summation of matching errors was reported as a cause of horizontal deviations [18].

Beside technology and device related errors there are also object inherent errors which contribute to the overall accuracy of the digital impression. Also the malocclusion has an effect on digital orthodontic models measurements. Significant differences in the amount of crowding due to the accumulation of errors were reported [20]. According to Bocklet et. al [3] the physical properties of the scanned tissues also play a major role in detecting surfaces. While problems concerning reflectivity and translucency are mainly solved, tissue movements in the oral cavity are still challenging. Tongue, soft palate, frenula and flexible mucosa are not detectable so most devices will stop scanning until hard tissue is detected again. There is no knowledge to which size of motion a scanner still produces error-free surfaces. Some movements like horizontal bending of the mandible during opening or tooth movement in the periodontal ligament on tongue pressure are below the software’s detection possibilities and contribute to the overall accuracy.

In Germany the statuary health insurances do not accept io-scans on legal grounds. There is a judicial decision that the impression must be connected with the production of the models. IOS do not meet the requirements for invoicing. This could be different in private insurances or other countries.

## 5.Conclusion

Dimensional differences between intraoral scans and conventional alginate impressions in the mixed dentition are clinically tolerable. In all clinical situations of active treatment in the mixed dentition, the intraoral scans are more detailed and less error-prone.

## Supplementary information

**Additional file 1: Table.** P-values of scan time (minutes:seconds) and amount of data (number of polygon points) concerning different patient variables.

## Data Availability

Datasets obtained or analysed during the current study are available from the corresponding author on reasonable request.
