# Supplementary material for: Accuracy of intraoral scans in the mixed dentition: a prospective non-randomized comparative clinical trial
Source: Head Face Med. 2020 May 19;16:11. doi: 10.1186/s13005-020-00222-6 (PMC7236363; doi:10.1186/s13005-020-00222-6)
Supplement: Supplementary file 1 — Additional file 1: Table. P-values of scan time (minutes:seconds) and amount of data (number of polygon points) concerning different patient variables. [file 13005_2020_222_MOESM1_ESM.pdf]

Supplementary table. P-values of scan time (minutes:seconds) and amount of data (number of polygon points) concerning different patient variables.

	**scan time**<br>*p-value*	**n of polygon points**<br>*p-value*
Gender	*0.2029*	*0.5877*
Dentition phase	*0.2989*	*0.0149*
Jaw	*0.1355*	*< 0.0001*
Angle class	*0.3305*	*0.4884*

P-values from Mann-Whitney U test or the Kruskal-Wallis test
